# Mass media promotion of a smartphone smoking cessation app: modelled health and cost-saving impacts

**DOI:** 10.1186/s12889-019-6605-8

**Published:** 2019-03-08

**Authors:** Nhung Nghiem, William Leung, Christine Cleghorn, Tony Blakely, Nick Wilson

**Affiliations:** 1 Department of Public Health, University of Otago, Wellington, New Zealand; 2 Dunedin School of Medicine, University of Otago, Dunedin, New Zealand

**Keywords:** Smartphone apps, Smoking cessation, Cost-utility analysis, mHealth, Tobacco control, Mass media

## Abstract

**Background:**

Smartphones are increasingly available and some high quality apps are available for smoking cessation. However, the cost-effectiveness of promoting such apps has never been studied. We therefore aimed to estimate the health gain, inequality impacts and cost-utility from a five-year promotion campaign of a smoking cessation smartphone app compared to business-as-usual (no app use for quitting).

**Methods:**

A well-established Markov macro-simulation model utilising a multi-state life-table was adapted to the intervention (lifetime horizon, 3% discount rate). The setting was the New Zealand (NZ) population (*N* = 4.4 million). The intervention effect size was from a multi-country randomised trial: relative risk for quitting at 6 months = 2.23 (95%CI: 1.08 to 4.77), albeit subsequently adjusted to consider long-term relapse. Intervention costs were based on NZ mass media promotion data and the NZ cost of attracting a smoker to smoking cessation services (NZ$64 per person).

**Results:**

The five-year intervention was estimated to generate 6760 QALYs (95%UI: 5420 to 8420) over the remaining lifetime of the population. For Māori (Indigenous population) there was 2.8 times the per capita age-standardised QALY gain relative to non-Māori. The intervention was also estimated to be cost-saving to the health system (saving NZ$115 million [m], 95%UI: 72.5m to 171m; US$81.8m). The cost-saving aspect of the intervention was maintained in scenario and sensitivity analyses where the discount rate was doubled to 6%, the effect size halved, and the intervention run for just 1 year.

**Conclusions:**

This study provides modelling-level evidence that mass-media promotion of a smartphone app for smoking cessation could generate health gain, reduce ethnic inequalities in health and save health system costs. Nevertheless, there are other tobacco control measures which generate considerably larger health gains and cost-savings such as raising tobacco taxes.

**Electronic supplementary material:**

The online version of this article (10.1186/s12889-019-6605-8) contains supplementary material, which is available to authorized users.

## Background

Smoking is the second most important risk factor for health loss globally according to the Global Burden of Disease Study [1]. In 2017 it caused 7·1 million deaths and the loss of 182 million disability-adjusted life years). Many evidence-based tobacco control interventions exist and new ones are continuing to emerge. One such novel approach is the use of mobile phone-based interventions for smoking cessation. These have been shown to be effective, as per a Cochrane systematic review [2] and a meta-analysis [3]. Less research has been performed around the use of smoking cessation apps on smartphones. Nevertheless, a review of eight studies of such apps indicated favourable quit rates for app users in the range of 13 to 24% [4]. Others subsequent studies have also reported favourable results [5, 6], but the first full randomised controlled trials (RCT) were not published until 2018. One of these was a multi-country study (Australia, Singapore, United Kingdom [UK] and the Unites States [US]), that reported a quit rate at 6 months of 8.5% in the intervention group vs 3.8% in the control group (a relative risk [RR] = 2.23; 95%CI: 1.08 to 4.77; intention-to-treat analysis) [7]. A particular strength of this study was the comparison of a “state-of-the-art” decision-aid design versus support with passive information-only apps. Another RCT was conducted in Canada with 19 to 29 year-olds, and used a printed self-help guide for the control group [8]. It reported continuous abstinence at 6 months was not significantly different at 7.8% for the smartphone app versus 9.2% the self-help guide (odds ratio = 0.83, 95% confidence interval [CI] 0.59–1.18). A third RCT was conducted in the US and involved mindfulness training via a smartphone app with experience sampling vs a control of experience sampling only [9] (this experience sampling involved a component of the app which queried smoking, craving, and mindfulness in real time). It reported no group difference in smoking abstinence at 6 months (9.8% vs 12.1% in the two groups respectively, *p* = 0.51). But within the intervention group, the relationship between craving and cigarettes per day decreased as treatment completion increased (*p* = 0.04).

Promotion of smoking cessation using mass media campaigns (and more targeted advertising) has been found to be a cost-effective investment in tobacco control [10, 11]. In New Zealand (NZ), such mass media campaigns have also been reported to be cost-effective when promoting the national quitline service [12]. This suggested to us the possible value of promotion of smoking apps as an additional intervention for those smokers not using the quitline. Therefore we aimed in this modelling study to estimate the health gains and impact on health costs of this particular approach to tobacco control in New Zealand. This is a high-income country with a national Smokefree Goal for 2025 [13]. It is also a country where tobacco control has a very large potential for health gain and cost-savings (eg, in one modelling study: 282,000 QALYs gained and NZ$5.4 billion in cost savings for a sinking lid intervention on tobacco supplies [14]). Furthermore differences in tobacco use [15] is a major contributor to health inequalities, especially between Māori (Indigenous population) and non-Māori New Zealanders [16].

## Methods

### Modelled intervention

Our modelled intervention consisted of promotion of the same particular app as used in the multi-country RCT [7], “Quit Advisor Plus” which is available for downloading free of charge from the Apple App Store. We assumed that for Android smartphone users, a similar quality and free smartphone app would be available, given our published survey of the quality of free smoking cessation apps available to New Zealand citizens on both Apple and Android platforms [17].

We assumed that these apps would be promoted on New Zealand health agency websites eg, the Ministry of Health website. The website promotion was assumed to be at the level of $72,000 per annum, which is the amount spent on this purpose by the New Zealand Health Promotion Agency on its “Breakfast eaters campaign” (see Additional file 1: Table S1). The apps would also be promoted with annual mass media promotion which was assumed to cost NZ$2,791,000 (the equivalent of the New Zealand Quitline service’s marketing budget) [18].

### Modelling approach

In modelling health gain and net health system costs we used a well-established Markov macro-simulation model utilizing a multi-state life-table approach: the “BODE^3^ Tobacco Model” including probabilistic uncertainty about multiple input parameters [12, 19–23]. This model includes 16 tobacco-related diseases using national data by sex, age and ethnicity for the whole New Zealand population in 2011. It uses a health system perspective and estimates quality-adjusted life-years (QALYs) gained and net health system costs over the remainder of the population’s lifetime with both discounted at 3%. We also conducted sensitivity and scenario analyses around the discount rate, the effect size, impact for Māori (Indigenous population) and how many years the intervention was run for.

### Intervention effect size

There seemed too much heterogeneity in the design of the three published RCTs identified to combine the results in a meta-analysis. So we just selected the RCT which we considered the most appropriate for the New Zealand population ie, the multi-country one [7]. This RCT had a wider age range than the Canadian RCT and also the design of the control group was probably better than the Canadian trial (which used a printed self-help guide as opposed to another type of smartphone app in the multi-country trial). It would also be likely to have more general population appeal than the mindfulness app used in the RCT in the US. The intervention effect size in this selected trial was of a relative risk [RR] for prolonged abstinence at 6 months of 2.23 (95%CI: 1.08 to 4.77) [7], but we adjusted this downwards to produce a long-term cessation rate to account for subsequent relapse (including using data from two meta-analyses, see Additional file 1: Table S3). For simplicity, we assumed that only those who would have made an unaided cessation attempt download the app, ie, we assumed this was a different population from those who would tend to use the Quitline for quitting. Taking the 20–34-year-old smokers of European/other (non-Māori) ethnicity as an example, we estimated the annual net cessation rates for unaided quitting ie, at 3.1% (men) and 3.7% (women) (see Additional file 1: Figure S1, Tables S2 to S5). To this we applied the adjusted intervention effect size amongst those estimated to be using the app in this age-group (11.3%), giving permanent quit rates of 7.1% (men) and 10.4% (women) for those app users in this demographic group in each intervention year (see Additional file 1 for workings and results around quit rates for all the demographic groups eg, Māori ethnicity, 35-54y and 55 + y age-groups). This heterogeneity was also included in the comparator arm of the model, where we also estimated the baseline unaided quit rates (ie, those not using the Quitline who are the target and comparator population for this evaluation).

### Intervention costs

We used the average cost of attracting a Quitline caller to the New Zealand Quitline Service as a proxy for getting a person to download the smoking cessation app onto their smartphone (ie, NZ$64 per person [18]). This amount is fairly similar to the cost from a US study where mass media promotion was estimated to trigger app downloads at the advertising cost of ~NZ$70 per enrolee [24]. To estimate the number of smokers downloading the app in each year, we divided the total expenditure on website promotion and mass media promotion by this $64 per person figure. This gave an estimate of 44,700 New Zealand smokers ($2,863,000 [based on $72,000 + $2,791,000 above] / $64) who would be expected to download the app in response to the promotional activities. We included uncertainty around these estimates, along with age-variation in the download rate, albeit based on UK data for smoking cessation app downloads [25]. This gave download proportions by smoker age-group of: 11.3% for 20-34y; 9.0% for 35-54y; and 1.8% for 55 + y (see Additional file 1 for details).

## Results

The five-year promotion of smoking cessation apps was estimated to generate 6760 QALYs (95%UI: 5420 to 8420) over the lifetime of the population (Table 1). For Māori there was 2.5 times the per capita gain relative to the non-Māori (at 3.14 vs 1.25 per 1000 population respectively); or a 2.78 times difference when age-standardised (Table 1 and Additional file 1: Table S6). The intervention was also estimated to be cost-saving to the health system (saving NZ$115 million [m], 95%UI: 72.5m to 171m; US$81.8m (US$ for 2017). The overall cost-saving aspect of the intervention was maintained in all sensitivity and scenario analyses including where the effect size was halved (Additional file 1: Table S7), the discount rate varied, including up to 6% (Additional file 1: Tables S8 and S9), and the intervention run for just one year (Additional file 1: Table S10). Other scenario analyses included running the intervention for 10 years and for 20 years (the latter yielding 19,600 QALYs gained and $418m in cost-savings, Additional file 1: Tables S10 and S11). The key driver of uncertainty for both the health gain and cost-savings was the relative risk for net annual cessation rates comparing those who used the smartphone app to those who quit unaided (as per the tornado plots: Additional file 1: Figure S2 to S5).

**Table 1:** Health gain (QALYs) and health system cost-savings from the promotion of a smartphone app for smoking cessation (base case: effect size from a RCT, five-year intervention period, 3% discounting; 95% uncertainty interval shown in brackets)

Demographic group	Non-Māori QALYs gained	Māori QALYs gained	Ethnic groupings combined, QALYs gained	Ethnic groupings combined, cost-savings (NZ$ million)
Sex and age groups combined	4650 (3540 to 5910)	2120 (1590 to 2720)	6760 (5420 to 8420)	$115 (72.5 to 171)
Males, 15–24 years	379 (200 to 619)	175 (92.6 to 292)	554 (347 to 835)	$14.8 (7.43 to 25.9)
Males, 25–44 years	1220 (821 to 1690)	390 (262 to 543)	1610 (1170 to 2150)	$40.1 (25.1 to 58.9)
Males, 45–64 years	654 (422 to 954)	142 (88.7 to 215)	796 (548 to 1100)	$11.7 (5.47 to 19.8)
Males, 65+ years	39.3 (24.2 to 59.4)	3.15 (1.77 to 4.84)	42.5 (27.1 to 62.7)	$-2.12 (−1.81 to −2.37)
Males, all ages	2300 (1730 to 2980)	710 (530 to 926)	3010 (2370 to 3780)	$60.1 (36.9 to 89.7)^a^
Females, 15–24 years	407 (218 to 650)	292 (155 to 479)	700 (447 to 1020)	$13.8 (6.91 to 23.1)
Females, 25–44 years	1280 (865 to 1790)	813 (548 to 1120)	2090 (1550 to 2720)	$38.9 (24.9 to 57.6)
Females, 45–64 years	628 (397 to 910)	295 (182 to 447)	924 (660 to 1250)	$9.4 (3.96 to 16.4)
Females, 65+ years	39.4 (24.7 to 58.8)	5.78 (3.34 to 8.80)	45.2 (29.9 to 65.1)	$-2.71 (− 2.46 to − 2.94)
Females, all ages	2350 (1750 to 3050)	1410 (1050 to 1830)	3760 (2970 to 4690)	$55.1 (34.1 to 82.6)^a^
Per capita (QALYs /1000 people & $)^b^	1.25 (0.95 to 1.58)	3.14 (2.36 to 4.03)	1.54 (1.23 to 1.91)	$26.2 (16.5 to 38.9)

^a^The total costs also include the relatively minor health system costs experienced for the 0–14 year old group (who do not benefit from the intervention), and hence the displayed age-groups do not sum exactly to the total

^b^The ratios reported in the text for Māori vs non-Māori per capita gains, also includes those for the age-standardised results (which are slightly different from those in this table)

## Discussion

This modelling study suggests that the mass media promotion of a smartphone app and its use would be likely to generate overall health gains, favour greater per capita gains for the Indigenous population (Māori), and achieve cost-savings to the health sector. These findings are perhaps not surprising given the overall evidence for such apps being effective (see *Introduction*) and the evidence from a systematic review that mobile phone-based interventions [2] are effective. The cost finding is also consistent with a study showing that text messaging for smoking cessation is cost-saving for the health sector [26].

A strength of this modelling is that it is the first such study to consider the cost-utility of smoking cessation apps (not just text message interventions) and it uses a well-established tobacco control model containing detailed epidemiological and costing data. Nevertheless, there are various limitations with this modelling:

- The effect size was based on just a single RCT (with quitting measured at 6 months), albeit the RCT (out of the three published as of October 2018) to be considered the most relevant to the New Zealand population. However, we did a scenario analysis with half the effect size, which may better reflect the mixed outcome of these three trials.
- The baseline quit rate does not capture recent features of the tobacco control scene in New Zealand such as: the rise of e-cigarette use [27], the adoption of standardised tobacco packaging in New Zealand in 2018, and tobacco industry actions (eg, discount brands) that may undermine the ongoing annual tobacco tax increases used in New Zealand [28].
- No account was taken of potentially more efficient marketing strategies eg, via use of social media as per one study [29]. Similarly, no account was made of the potential synergies that could be achieved if app promotion was focused around the timing of World Smokefree Day activities or the annual rise in tobacco taxes in January of each year in New Zealand.
- The modelling only took a health system perspective. If a broader societal perspective was taken then the benefits would be higher since smoking is negatively related to long-term labour market outcomes [30].

### Potential implications for further research

Given the above issues, additional RCTs are desirable, especially those measuring quitting out to 12 months and actual costs of recruitment that might likely occur in population roll out. There is also a need to better understand smokers’ views on smoking cessation apps promoted by health authorities as opposed to the vast number of such apps already available online (many of questionable quality).

### Potential implications for policy-makers

In addition to supporting further research, policy-makers could consider comparing smoking cessation app promotion with other potential tobacco control interventions eg, as per the published league table of tobacco control studies using the “BODE^3^ Tobacco Model” in Nghiem et al. [12] and in an online interactive league table that includes Australian tobacco control interventions [31]. Some of these major interventions (eg, further tax increases, a tobacco-free generation and a sinking lid on supply) would be likely to generate much greater health gain (although they are also typically applied for greater than this five-year intervention, indeed lifelong), as well as accelerating progress to tobacco endgame goals. But if policy-makers favour smoking cessation app promotion as a necessary part of more comprehensive programmes, then they could consider initial moves such as investigating resources in monitoring the market for the best apps and promoting these on health agency websites (as is already done by the National Health Service in the UK [32]).

## Conclusions

This study provides modelling-level evidence that mass-media promotion of a smartphone app for smoking cessation is likely to generate health gain, reduce ethnic inequalities in health and save health system costs. The five-year intervention was estimated to generate 6760 QALYs and save NZ$ 73 million over the remaining lifetime of the population. Nevertheless, there are other tobacco control which generate considerably larger health gains and cost-savings such as raising tobacco taxes.

## Additional file

Additional file 1: **Table S1.** Summary of key tobacco model parameters. **Table S2.** Net annual cessation rates, quit attempt, and quitline use (%) for different population groups. **Figure S1.** Flow diagram of smokers in intervention arm (app use) and other cessation approaches. **Table S3.** Calculations of net annual cessation rates for the 20-34y age group (see subsequent tables for other age-groups, uncertainty not shown). **Table S4.** Calculations of net annual cessation rates for the 35-54y age group (see other tables for other age-groups, uncertainty not shown). **Table S5.** Calculations of net annual cessation rates for the 55+ year age-group group (see other tables for other age-groups, uncertainty not shown). **Figure S2.** Tornado plot for uncertainty around health gain (in QALYs) for the base-case analysis (3% discount rate) for non-Māori all age and both sexes. **Figure S3.** Tornado plot for uncertainty around cost-savings for the base-case analysis (3% discount rate) for non-Māori all age and both sexes. **Figure S4.** Tornado plot for uncertainty around health gain (in QALYs) for the base-case analysis (3% discount rate) for Māori all age and both sexes. **Figure S5.** Tornado plot for uncertainty around cost-savings for the base-case analysis (3% discount rate) for Māori all age and both sexes. **Table S6.** Base-case with Māori equity analysis (3% discount rate). **Table S7.** Scenario analysis with half the intervention effect size used in the base-case. **Table S8.** Sensitivity analysis with the discount rate set at 0%, otherwise the base-case. **Table S9.** Sensitivity analysis with the discount rate set at 6%, otherwise the base-case. **Table S10.** Health gain (QALYs) and cost-savings for all scenario and sensitivity analyses: All ages and sexes combined. **Table S11.** Health gain (QALYs) and cost-savings at the individual citizen level (including both smokers and non-smokers). (DOCX 99 kb)

## Abbreviations

CI: confidence interval
NZ: New Zealand
QALYs: quality-adjusted life-years
RCT: randomised controlled trial
UK: United Kingdom
US: United States
