# Supplementary material for: Mass media promotion of a smartphone smoking cessation app: modelled health and cost-saving impacts
Source: BMC Public Health. 2019 Mar 8;19:283. doi: 10.1186/s12889-019-6605-8 (PMC6408783; doi:10.1186/s12889-019-6605-8)
Supplement: Supplementary file 1 — Table S1. Summary of key tobacco model parameters. Table S2. Net annual cessation rates, quit attempt, and quitline use (%) for different population groups. Figure S1. Flow diagram of smokers in intervention arm (app use) and other cessation approaches. Table S3. Calculations of net annual cessation rates for the 20-34y age group (see subsequent tables for other age-groups, uncertainty not shown). Table S4. Calculations of net annual cessation rates for the 35-54y age group (see other tables for other age-groups, uncertainty not shown). Table S5. Calculations of net annual cessation rates for the 55+ year age-group group (see other tables for other age-groups, uncertainty not shown). Figure S2. Tornado plot for uncertainty around health gain (in QALYs) for the base-case analysis (3% discount rate) for non-Māori all age and both sexes. Figure S3. Tornado plot for uncertainty around cost-savings for the base-case analysis (3% discount rate) for non-Māori all age and both sexes. Figure S4. Tornado plot for uncertainty around health gain (in QALYs) for the base-case analysis (3% discount rate) for Māori all age and both sexes. Figure S5. Tornado plot for uncertainty around cost-savings for the base-case analysis (3% discount rate) for Māori all age and both sexes. Table S6. Base-case with Māori equity analysis (3% discount rate). Table S7. Scenario analysis with half the intervention effect size used in the base-case. Table S8. Sensitivity analysis with the discount rate set at 0%, otherwise the base-case. Table S9. Sensitivity analysis with the discount rate set at 6%, otherwise the base-case. Table S10. Health gain (QALYs) and cost-savings for all scenario and sensitivity analyses: All ages and sexes combined. Table S11. Health gain (QALYs) and cost-savings at the individual citizen level (including both smokers and non-smokers). (DOCX 99 kb) [file 12889_2019_6605_MOESM1_ESM.docx]

**Additional File 1: Additional Methods and Results**

# Additional methods

## Modelling and key parameters

Table S1: Summary of key tobacco model parameters

| **Input parameter** |  |  |  |
| --- | --- | --- | --- |
| **Epidemiological parameters** | **Source / details** | **Heterogeneity** | **Uncertainty** |
| Background model parameters for business-as-usual smoking trends in the NZ population combined with demographic and mortality trends | Blakely et al [1] | Variation by age/sex/ ethnicity (Table S2) | Blakely et al [1] and (Table S2) |
| Epidemiological data on 16 tobacco-related diseases and associated RRs by smoking status (current-, ex- and never-smoker) along with time lags | Blakely et al [1] | Variation by age/sex/ ethnicity (but not the RR values by ethnicity) | Blakely et al [1] |
| Disease morbidity rates per capita | For each of the 16 tobacco-related diseases, there was an assigned disability rate (DR; by sex and age) equal to YLDs for that disease (scaled down to adjust for comorbidities) from the 2006 NZBDS projected forward to 2011, divided by the disease prevalence (above). This DR was assigned to the proportion of the cohort in each disease state. No trends (ie, assumed constant into the future) [1]. | Variation by age/sex | ±10% SD, normal distribution |
| Total morbidity per capita in 2011 | The per capita rate of years of life lived with disability (YLD) was from the NZBDS by sex, age, and ethnicity. No trend (ie, assumed constant into the future). See elsewhere [1]. | Variation by age/sex/ ethnicity | ±10% SD, log-normal distribution |
| Estimated effectiveness of the package of mass media promotion of the smartphone apps in NZ (mass media promotion for 20y) | See Tables S3-S5 | Variation by age/sex/ ethnicity (see Tables S3-S5) | See Tables S3-S5 |
| ***Costs and app downloads generated*** |  |  |  |
| Background health system costs for all citizens and costs of the 16 tobacco-related diseases | Blakely et al [1] and HealthTracker [2]. An update on the costing approach is detailed elsewhere [3]. | Variation by age/sex (but not ethnicity) | Blakely et al [1] |
| Intervention cost (A): Nation-wide promotion of the smartphone apps to non-Quitline users | Annual mass media promotion was assumed to cost NZ$2,791,000 (the NZ Quitline service’s marketing budget) [4]. | Applied to the total population | See below |
| Intervention cost (B): Nation-wide promotion of the smartphone apps to non-Quitline users | The annual cost of promotion on relevant government-funded websites (Ministry of Health, District Health Boards, Health Promotion Agency) of NZ$72,000. This amount was based on the New Zealand Health Promotion Agency Breakfast eaters campaign [Personal Communication, HPA, October 2015] for online promotions (Google adwords, Facebook adverts, promoting Facebook posts etc) to drive consumers to the Breakfast-eaters website. | Applied to the total population | See below |
| Total intervention cost | NZ$2,863,000 per year (the sum of the above two rows) | Applied to the total population | ±10% SD, gamma distribution |
| App downloads generated | We used the average cost of attracting a Quitline caller to the Quitline service as a proxy for getting a person to download the smoking cessation app onto their smartphone. That is NZ$64 per person [4], which is an amount that is fairly similar to the cost from a US study where mass media promotion was estimated to trigger app downloads at the advertising cost of ~NZ$70 per enrolee [5]. This meant that there were estimated to be 44,700 New Zealand smokers who downloaded the app ($2,863,000/$64). | The proportions of downloads varied by age-group: 50.4% were amongst ages 20-34y; 45.4% were amongst ages 35-54y; and 4.2% were amongst ages 55+y (based on UK data for smoking cessation app downloads [6]. | Not considered |

Net annual smoking cessation rate data for different population groups (${NCR}_{p,C}$) were obtained from a study that examined smoking trends from census data (which collects smoking data), as per Table S2. These “net” rates reflect long-term cessation outcomes, and so incorporate the fact that many smokers will quit and relapse multiple times before finally succeeding.

Table S2. Net annual cessation rates, quit attempt, and quitline use (%) for different population groups

| **Age-group (years)** | **Non-Māori smokers** | | **Māori smokers** | |
| --- | --- | --- | --- | --- |
|  | **Men** | **Women** | **Men** | **Women** |
| Net annual cessation rates (${NCR}_{p,C}$) based on a comparison of 2006 and 2013 Census data [7] (95%UIs in parentheses) | | | | |
| 20-34 | 4.1 (4.0 to 4.3) | 5.5 (5.3 to 5.8) | 3.9 (3.7 to 4.2) | 4.5 (4.2 to 4.8) |
| 35-54 | 3.8 (3.7 to 4.0) | 4.3 (4.1 to 4.5) | 3.7 (3.5 to 3.9) | 4.7 (4.5 to 5.0) |
| 55+ | 7.2 (7.0 to 7.5) | 7.1 (6.9 to 7.4) | 7.7 (7.1 to 8.3) | 6.9 (6.4 to 7.6) |
| Percentage of smokers reporting trying to quit in the last year (for >24h, daily smokers) ($a_{p}$*%*) using NZ Health Survey (NZHS) 2012/13 data.[8]* In the analyses, 95%UIs were estimated at +/- 5% points relative to the point estimate, normal distribution. | | | | |
| 20-34 | 69.6 | 73.0 | 57.8 | 60.7 |
| 35-54 | 66.4 | 66.3 | 55.2 | 55.1 |
| 55+ | 60.7 | 69.1 | 50.4 | 57.3 |
| Percentage of smokers who stated that they used the quitline on the last quit attempt ($b_{p}$*%*) using NZHS 2012/13 data [8].* In the analyses, 95%UIs were estimated at +/- 3% points relative to the point estimate, normal distribution). | | | | |
| 20-34 | 13.0 | | 19.0 | |
| 35-54 | 9.7 | | 14.3 | |
| 55+ | 10.0 | | 14.7 | |

* NZHS data were combined into these larger age-groups (with adjustments to give ethnicity specific values – as these were not given by age-group). There were no data on sex distribution for quitline use.

The values in the first three rows of Table S2 reflect the impact of the comparator – business-as-usual quitline and also non-quitline related cessation. It is necessary to estimate the latter as the smoking cessation app intervention will be specifically targeted at non-quitline users. Thus we considered the following equations where:

${NCR}_{p,C}$ the actual observed net annual cessation rate in the specific population group *p* (as per Table S2 by age/sex/ethnic group) in the comparator arm.*

${NCR}_{p,I}$ the estimated net annual cessation rate in the specific population group *p* (by age/sex/ethnic group) in the intervention arm.*

${NCR}_{p,Q}$ the net annual cessation rate for those who call the quitline (in each specific population *p* by age/sex/ethnic group).*

${NCR}_{p,U}$ the net annual cessation rate for those who don’t call the quitline (in each specific population *p* by age/sex/ethnic group); these people quit as a result of other interventions – “unaided” cessation.*

${NCR}_{p,A}$ the net annual cessation rate for those who use a smartphone smoking cessation app (in each specific population *p* by age/sex/ethnic group).*

$P_{p,Q}$ the proportion of the smoker population who call the quitline (in each specific population *p* by age/sex/ethnic group).

$P_{p,A}$ the proportion of the smoker population who use a smoking cessation app (in each specific population *p* by age/sex/ethnic group).

${RR}_{A}$ the relative risk for net annual cessation rates, comparing smartphone app use to unaided smoking cessation (${NCR}_{p,U}$). The ${RR}_{A}$ of 2.23 (95%CI: 1.08 to 4.77) from the available RCT (see rationale in the main manuscript).

* That is the number of complete long-term quitters in a year in this group, over the prevalent population of smokers (in that age/sex/ethnic group).

The net annual cessation rate in the specific population groups is defined in equation 1 below

${NCR}_{p,C}=P_{p,Q}\times{NCR}_{p,Q}+(1-P_{p,Q})\times{NCR}_{p,U}$ (eq 1)

Solving for ${NCR}_{p,U}$

${NCR}_{p,U}=\frac{\left( {NCR}_{p,C}-\left[ P_{p,Q}\times{NCR}_{p,Q} \right] \right)}{\left( 1-P_{p,Q} \right)}$ (eq 2)

Net annual cessation rate for those who use a smartphone smoking cessation app was estimated thus

${NCR}_{p,A}={RR}_{A}\times{NCR}_{p,U}$ (eq 3)

The quit rate in the intervention arm was modelled through an increase in net annual cessation rate in the year of intervention for the percentage ($P_{p,A}$) of the specific demographic group downloading the app that year.

${NCR}_{p,I}= {NCR}_{p,C}+\left( P_{p,A}\times\left[ {NCR}_{p,A}-{NCR}_{p,U} \right] \right)$ (eq 4)

A simplified flow diagram summarizing how each specific population group, of the 2011 NZ population of smokers, can annually transition through the various smoking cessation options in the intervention arm is shown below.

${NCR}_{p,U}$

${NCR}_{p,A}$

${NCR}_{p,Q}$

If trying to quit this year, choose a method

2011 NZ population of smokers at year *y*

Use Quitline

Download/ use app

Attempt “unaided” cessation

Ex-smokers who have reduced risk of 16 tobacco-related diseases

Figure S1: Flow diagram of smokers in intervention arm (app use) and other cessation approaches

Table S3: Calculations of net annual cessation rates for the 20-34y age group (see subsequent tables for other age-groups, uncertainty not shown)

| **Key parameters for the 20-34y age group** | **Non-Māori smokers** | | **Māori  smokers** | |
| --- | --- | --- | --- | --- |
|  | **Men** | **Women** | **Men** | **Women** |
| 1. Net cessation rate in the comparator arm, $\boldsymbol{NCR}_{\boldsymbol{p,C}}$ from Table S2. | 4.1% | 5.5% | 3.9% | 4.5% |
| 2. Daily smokers reporting trying to quit (for >24h) in the last year using NZHS 2012/13 Excel data [8]. Variable $\boldsymbol{a}_{\boldsymbol{p}}$ from Table S2. | 69.6% | 73.0% | 57.8% | 60.7% |
| 3. Proportion of smokers who stated they used the Quitline on the last quit attempt from NZHS 2012/13 Excel data [8]. Variable $\boldsymbol{b}_{\boldsymbol{p}}$ from Table S2. | 13% | | 19% | |
| 4. The proportion of smokers who use the Quitline, $\boldsymbol{P}_{\boldsymbol{p,Q}}$= $a_{p}\times b_{p}$ | 9.05% | 9.49% | 10.98% | 11.53% |
| 5. Quitting success rate of using the Quitline [4] at six months (2011 data, no ethnic/sex-specific data reported). | 24.2% quit at 6 months | | | |
| 6. Net cessation rate for quitline users, $\boldsymbol{NCR}_{\boldsymbol{p,Q}}$. Estimates around further attenuation of quitting success were based on meta-analysis results for relapse between the 6-month and 12-month points (21% from 6 to 12 months; 95%UI 19 to 24%) [9]. Then for cumulative post 12-month relapse we used results from another meta-analysis (relapse of 30% by 2 years; 95%UI 23.5 to 37.5%) [10]. | 24.2% $\times$ (100% - 21%) $\times$ (100% - 30%)  = 13.4% permanently quit in the long-term  We assumed a SD = ±20%, normal distribution. | | | |
| 7. Net cessation rate for unaided cessation, $\boldsymbol{NCR}_{\boldsymbol{p,U}}$ as per eq 2. | 3.18% | 4.67% | 2.73% | 3.34% |
| 8. The proportion of smokers who, after mass promotion, download or use a smoking cessation app, $\boldsymbol{P}_{\boldsymbol{p,A}}$.  Based on a proportion of the 44,700 New Zealander who are estimated to download the app (see Methods). It is assumed that 50.4% of smoking cessation app users are in this age group [6] and there is no difference in usage by ethnicity or sex. | 11.3% (uncertainty was SD = +/- 10%) | | | |
| 9. Net cessation rate for smartphone app users, $\boldsymbol{NCR}_{\boldsymbol{p,A}}$ as per eq 3. | 7.09% | 10.41% | 6.09% | 7.45% |
| 10. Net cessation rate in the intervention arm, $\boldsymbol{NCR}_{\boldsymbol{p,I}}$ as per eq 4. | 4.54% | 6.15% | 4.28% | 4.96% |

Table S4: Calculations of net annual cessation rates for the 35-54y age group (see other tables for other age-groups, uncertainty not shown)

| **Key parameters for the 35-54y age group** | **Non-Māori smokers** | | **Māori  smokers** | |
| --- | --- | --- | --- | --- |
|  | **Men** | **Women** | **Men** | **Women** |
| 1. Net cessation rate in the comparator arm, $\boldsymbol{NCR}_{\boldsymbol{p,C}}$ from Table S2. | 3.8% | 4.3% | 3.7% | 4.7% |
| 2. Daily smokers reporting trying to quit (for >24h) in the last year using NZHS 2012/13 Excel data [8]. Variable $\boldsymbol{a}_{\boldsymbol{p}}$ from Table S2. | 66.4% | 66.3% | 55.2% | 55.1% |
| 3. Proportion of smokers who stated they used the Quitline on the last quit attempt from NZHS 2012/13 Excel data [8]. Variable $\boldsymbol{b}_{\boldsymbol{p}}$ from Table S2. | 9.7% | | 14.3% | |
| 4. The proportion of smokers who use the Quitline, $\boldsymbol{P}_{\boldsymbol{p,Q}}$= $a_{p}\times b_{p}$ | 6.44% | 6.43% | 7.89% | 7.88% |
| 5. Quitting success rate of using the Quitline [4] at six months (2011 data, no ethnic/sex-specific data reported). | 24.2% quit at 6 months | | | |
| 6. Net cessation rate for quitline users, $\boldsymbol{NCR}_{\boldsymbol{p,Q}}$. Derivation same for all age groups (see row 6 in  Table S3). | 13.4% permanently quit in the long-term,  we assumed a SD = ±20%, normal distribution. | | | |
| 7. Net cessation rate for unaided cessation, $\boldsymbol{NCR}_{\boldsymbol{p,U}}$ as per eq 2. | 3.14% | 3.68% | 2.87% | 3.96% |
| 8. The proportion of smokers who, after mass promotion, download or use a smoking cessation app, $\boldsymbol{P}_{\boldsymbol{p,A}}$.  Based on a proportion of the 44,700 New Zealander who are estimated to download the app (see Methods). It is assumed that 45.4% of smoking cessation app users are in this age group [6] and there is no difference in usage by ethnicity or sex. | 11.3% (uncertainty was SD = +/- 10%) | | | |
| 9. Net cessation rate for smartphone app users, $\boldsymbol{NCR}_{\boldsymbol{p,A}}$ as per eq 3. | 7.00% | 8.21% | 6.40% | 8.83% |
| 10. Net cessation rate in the intervention arm, $\boldsymbol{NCR}_{\boldsymbol{p,I}}$ as per eq 4. | 4.15% | 4.71% | 4.02% | 5.14% |

Table S5: Calculations of net annual cessation rates for the 55+ year age-group group (see other tables for other age-groups, uncertainty not shown)

| **Key parameters for the 55+y age group** | **Non-Māori smokers** | | **Māori  smokers** | |
| --- | --- | --- | --- | --- |
|  | **Men** | **Women** | **Men** | **Women** |
| 1. Net cessation rate in the comparator arm, $\boldsymbol{NCR}_{\boldsymbol{p,C}}$ from Table S2. | 7.2% | 7.1% | 7.7% | 6.9% |
| 2. Daily smokers reporting trying to quit (for >24h) in the last year using NZHS 2012/13 Excel data [8]. Variable $\boldsymbol{a}_{\boldsymbol{p}}$ from Table S2. | 60.7% | 69.1% | 50.4% | 57.3% |
| 3. Proportion of smokers who stated they used the Quitline on the last quit attempt from NZHS 2012/13 Excel data [8]. Variable $\boldsymbol{b}_{\boldsymbol{p}}$ from Table S2. | 10.0% | | 14.7% | |
| 4. The proportion of smokers who use the Quitline, $\boldsymbol{P}_{\boldsymbol{p,Q}}$= $a_{p}\times b_{p}$ | 6.07% | 6.91% | 7.41% | 8.42% |
| 5. Quitting success rate of using the Quitline [4] at six months (2011 data, no ethnic/sex-specific data reported). | 24.2% quit at 6 months | | | |
| 6. Net cessation rate for quitline users, $\boldsymbol{NCR}_{\boldsymbol{p,Q}}$. Derivation same for all age groups (see row 6 in  Table S3). | 13.4% permanently quit in the long-term,  we assumed a SD = ±20%, normal distribution. | | | |
| 7. Net cessation rate for unaided cessation, $\boldsymbol{NCR}_{\boldsymbol{p,U}}$ as per eq 2. | 6.80% | 6.63% | 7.25% | 6.30% |
| 8. The proportion of smokers who, after mass promotion, download or use a smoking cessation app, $\boldsymbol{P}_{\boldsymbol{p,A}}$.  Based on a proportion of the 44,700 New Zealander who are estimated to download the app (see Methods). It is assumed that 4.2% of smoking cessation app users are in this age group [6] and there is no difference in usage by ethnicity or sex. | 1.8% (uncertainty was SD = +/- 10%) | | | |
| 9. Net cessation rate for smartphone app users, $\boldsymbol{NCR}_{\boldsymbol{p,A}}$ as per eq 3. | 15.16% | 14.78% | 16.17% | 14.05% |
| 10. Net cessation rate in the intervention arm, $\boldsymbol{NCR}_{\boldsymbol{p,I}}$ as per eq 4. | 7.35% | 7.25% | 7.86% | 7.04% |

# Additional results

## Sensitivity and scenario analyses

The results in Figures S2 to S5 show that RR_A_, the relative risk for net annual cessation rates comparing those who use a smartphone app to those who quit unaided, is the single variable that causes the most uncertainty in both QALYs gained per year per 1000 population and incremental cost-savings per capita.

Figure S2: Tornado plot for uncertainty around health gain (in QALYs) for the base-case analysis (3% discount rate) for non-Māori all age and both sexes

RR_A_ the relative risk for net annual cessation rates, comparing those who use a smartphone app to those who quit unaided (ie, who don’t call the Quitline);

NCR_p,U_ the net annual cessation rate for those who quit unaided;

NCR_p,C_  the net annual cessation rate in the specific population groups in the comparator arm;

a_p_ smokers reporting trying to quit in the last year;

b_p_ proportion of smokers who stated that they used the Quitline on the last quit attempt

Figure S3: Tornado plot for uncertainty around cost-savings for the base-case analysis (3% discount rate) for non-Māori all age and both sexes

RR_A_ the relative risk for net annual cessation rates, comparing those who use a smartphone app to those who quit unaided (ie, who don’t call the Quitline);

COPD costs – cost of chronic obstructive pulmonary disease (COPD) to the health system;

NCR_p,U_ the net annual cessation rate for those who quit unaided;

Citizen cost – average citizen annual health costs (not in the last six months of life and not concurrently alive with one of the modelled tobacco-related diseases);

NCR_p,C_  the net annual cessation rate in the specific population groups in the comparator arm;

Lung cancer costs – cost of lung cancer to the health system;

a_p_ smokers reporting trying to quit in the last year;

b_p_ proportion of smokers who stated that they used the Quitline on the last quit attempt.

Figure S4: Tornado plot for uncertainty around health gain (in QALYs) for the base-case analysis (3% discount rate) for Māori all age and both sexes

RR_A_ the relative risk for net annual cessation rates, comparing those who use a smartphone app to those who quit unaided (ie, who don’t call the Quitline);

NCR_p,U_ the net annual cessation rate for those who quit unaided;

NCR_p,C_  the net annual cessation rate in the specific population groups in the comparator arm;

a_p_ smokers reporting trying to quit in the last year;

b_p_ proportion of smokers who stated that they used the Quitline on the last quit attempt

| Figure S5: Tornado plot for uncertainty around cost-savings for the base-case analysis (3% discount rate) for Māori all age and both sexes |
| --- |
|  |
|  |
|  |
|  |

RR_A_ the relative risk for net annual cessation rates, comparing those who use a smartphone app to those who quit unaided (ie, who don’t call the Quitline);

COPD costs – cost of chronic obstructive pulmonary disease (COPD) to the health system;

NCR_p,U_ the net annual cessation rate for those who quit unaided;

Citizen cost – average citizen annual health costs (not in the last 6six months of life and not concurrently alive with one of the modelled tobacco-related diseases);

NCR_p,C_  the net annual cessation rate in the specific population groups in the comparator arm;

Lung cancer costs – cost of lung cancer to the health system;

a_p_ smokers reporting trying to quit in the last year;

b_p_ proportion of smokers who stated that they used the Quitline on the last quit attempt.

## Sensitivity and scenario analyses

Table S6: Base-case with Māori equity analysis* (3% discount rate)

| **Demographic group** | **Non-Māori QALYs gained** | **Māori QALYs gained** | **Ethnic groupings combined, QALYs gained** | **Ethnic groupings combined, cost-savings (NZ$ million)** |
| --- | --- | --- | --- | --- |
| **Sex and age groups combined** | 4640 | 2300 | 6950 | $115 |
| Males, 15-24 year olds | 377 | 189 | 566 | $14.8 |
| Males, 25-44 year olds | 1230 | 430 | 1660 | $40.3 |
| Males, 45-64 year olds | 650 | 159 | 809 | $11.6 |
| Males, 65+ year olds | 39.4 | 3.68 | 43.1 | $-2.12 |
| **Males, all ages** | 2290 | 782 | 3080 | $60.2 |
| Females, 15-24 year olds | 406 | 312 | 718 | $13.8 |
| Females, 25-44 year olds | 1280 | 879 | 2160 | $39.0 |
| Females, 45-64 year olds | 625 | 323 | 948 | $9.3 |
| Females, 65+ year olds | 39.6 | 6.62 | 46.2 | $-2.71 |
| **Females, all ages** | 2350 | 1520 | 3870 | $55.3 |
| Per capita (QALYs /1000 people and $) | 1.24 | 3.42 | 1.58 | $26.2 |

* In this analysis we give the Māori population the same potential envelope of health gain as per non-Māori, ie, the same morbidity and mortality rates as non-Māori [11].

Table S7: Scenario analysis with half the intervention effect size used in the base-case

| **Demographic group** | **Non-Māori QALYs gained** | **Māori QALYs gained** | **Ethnic groupings combined, QALYs gained** | **Ethnic groupings combined, cost-savings (NZ$ million)** |
| --- | --- | --- | --- | --- |
| **Sex and age groups combined** | 2330 | 1070 | 3400 | $36.7 |
| Males, 15-24 year olds | 189 | 87.6 | 277 | $5.81 |
| Males, 25-44 year olds | 616 | 197 | 814 | $17.5 |
| Males, 45-64 year olds | 326 | 71.8 | 398 | $3.21 |
| Males, 65+ year olds | 19.7 | 1.61 | 21.4 | $-2.36 |
| **Males, all ages** | 1150 | 358 | 1510 | $19.7 |
| Females, 15-24 year olds | 204 | 147 | 351 | $5.45 |
| Females, 25-44 year olds | 641 | 411 | 1050 | $16.7 |
| Females, 45-64 year olds | 313 | 149 | 463 | $1.93 |
| Females, 65+ year olds | 19.8 | 2.96 | 22.8 | $-2.90 |
| **Females, all ages** | 1180 | 710 | 1890 | $16.9 |
| Per capita (QALYs /1000 people and $) | 0.624 | 1.58 | 0.771 | $8.33 |

Table S8: Sensitivity analysis with the discount rate set at 0%, otherwise the base-case

| **Demographic group** | **Non-Māori QALYs gained** | **Māori QALYs gained** | **Ethnic groupings combined, QALYs gained** | **Ethnic groupings combined, cost-savings (NZ$ million)** |
| --- | --- | --- | --- | --- |
| **Sex and age groups combined** | 15,900 | 7060 | 22,900 | $327 |
| Males, 15-24 year olds | 1810 | 781 | 2600 | $54.4 |
| Males, 25-44 year olds | 4350 | 1280 | 5630 | $102 |
| Males, 45-64 year olds | 1620 | 330 | 1950 | $22.0 |
| Males, 65+ year olds | 68.6 | 5.26 | 73.9 | $-2.83 |
| **Males, all ages** | 7850 | 2400 | 10,300 | $170 |
| Females, 15-24 year olds | 1910 | 1310 | 3230 | $49.9 |
| Females, 25-44 year olds | 4470 | 2640 | 7110 | $98.0 |
| Females, 45-64 year olds | 1570 | 694 | 2260 | $18.2 |
| Females, 65+ year olds | 69.8 | 9.9 | 79.7 | $-3.58 |
| **Females, all ages** | 8020 | 4660 | 12,700 | $157 |
| Per capita (QALYs /1000 people and $) | 4.25 | 10.5 | 5.21 | $74.3 |

Table S9: Sensitivity analysis with the discount rate set at 6%, otherwise the base-case

| **Demographic group** | **Non-Māori QALYs gained** | **Māori QALYs gained** | **Ethnic groupings combined, QALYs gained** | **Ethnic groupings combined, cost-savings (NZ$ million)** |
| --- | --- | --- | --- | --- |
| **Sex and age groups combined** | 1640 | 765 | 2410 | $38.9 |
| Males, 15-24 year olds | 95.6 | 46.4 | 142 | $3.55 |
| Males, 25-44 year olds | 405 | 139 | 543 | $16.3 |
| Males, 45-64 year olds | 286 | 67.6 | 354 | $5.89 |
| Males, 65+ year olds | 23.7 | 2.05 | 25.7 | $-1.67 |
| **Males, all ages** | 810 | 255 | 1060 | $20.5 |
| Females, 15-24 year olds | 108 | 77.4 | 185 | $3.42 |
| Females, 25-44 year olds | 429 | 291 | 720 | $15.9 |
| Females, 45-64 year olds | 273 | 139 | 411 | $4.50 |
| Females, 65+ year olds | 23.4 | 3.66 | 27.1 | $-2.14 |
| **Females, all ages** | 833 | 510 | 1340 | $18.4 |
| Per capita (QALYs /1000 people and $) | 0.440 | 1.13 | 0.547 | $8.84 |

Table S10: Health gain (QALYs) and cost-savings for all scenario and sensitivity analyses: All ages and sexes combined

| **Scenario / sensitivity analysis** | **Non-Māori QALYs gained** | **Māori QALYs gained** | **Ethnic groupings combined, QALYs gained** | **Ethnic groupings combined, cost-savings (NZ$ million)** |
| --- | --- | --- | --- | --- |
| Baseline (3% discount rate, 5y intervention) | 4650 | 2120 | 6760 | $115 |
| Baseline with Māori equity | 4640 | 2300 | 6950 | $115 |
| Baseline – but with a time horizon for health and cost impacts of just 2011 to 2030 (rather than lifetime) | – | – | 760 | $2.81 |
| Half effect size (otherwise baseline) | 2330 | 1070 | 3400 | $36.7 |
| 0% discount rate (otherwise baseline) | 15,900 | 7060 | 22,900 | $327 |
| 6% discount rate (otherwise baseline) | 1640 | 765 | 2410 | $38.9 |
| Intervention only implemented for: 1y year (otherwise baseline) | 1060 | 459 | 1520 | $32.4 |
| - 10 years | 8160 | 3960 | 12,100 | $241 |
| - 20 years | 12,800 | 6880 | 19,600 | $418 |

Table S11: Health gain (QALYs) and cost-savings at the individual citizen level (including both smokers and non-smokers)

| **Scenario / sensitivity analysis** | **Non-Māori QALYs/1000 people** | **Māori QALYs/1000 people** | **Ethnic groupings combined, QALYs/1000 people** | **Ethnic groupings combined, cost-savings (NZ$)/1000 people** |
| --- | --- | --- | --- | --- |
| Baseline (3% discount rate, 5y intervention) | 1.25 | 3.14 | 1.54 | $26.2 |
| Baseline with Māori equity | 1.24 | 3.42 | 1.58 | $26.2 |
| Half effect size (otherwise baseline) | 0.624 | 1.58 | 0.771 | $8.33 |
| 0% discount rate (otherwise baseline) | 4.25 | 10.5 | 5.21 | $74.3 |
| 6% discount rate (otherwise baseline) | 0.440 | 1.13 | 0.547 | $8.84 |
| Intervention only implemented for: 1y year (otherwise baseline) | 0.283 | 0.681 | 0.344 | $7.36 |
| - 10 years | 2.19 | 5.87 | 2.75 | $54.7 |
| - 20 years | 3.42 | 10.2 | 4.46 | $94.8 |

# References

1. Blakely T, Cobiac LJ, Cleghorn CL, Pearson AL, van der Deen FS, Kvizhinadze G, Nghiem N, McLeod M, Wilson N. Correction: Health, Health Inequality, and Cost Impacts of Annual Increases in Tobacco Tax: Multistate Life Table Modeling in New Zealand. PLoS Med*.* 2016;13(12):e1002211.

2. Blakely T, Atkinson J, Kvizhinadze G, Nghiem N, McLeod H, Davies A, Wilson N. Updated New Zealand health system cost estimates from health events by sex, age and proximity to death: further improvements in the age of ‘big data’. N Z Med J*.* 2015;128(1422):13-23.

3. Pearson AL, Cleghorn CL, van der Deen FS, Cobiac LJ, Kvizhinadze G, Nghiem N, Blakely T, Wilson N. Tobacco retail outlet restrictions: health and cost impacts from multistate life-table modelling in a national population. Tob Control*.* 2016;(E-publication 22 September).

4. Quitline: Quitline Me Mutu Annual Review 2013/2014. Wellington: Quitline; 2014.

5. Buller DB, Borland R, Bettinghaus EP, Shane JH, Zimmerman DE. Randomized trial of a smartphone mobile application compared to text messaging to support smoking cessation. Telemedicine J e-health*.* 2014;20(3):206-214.

6. Ubhi HK, Michie S, Kotz D, Wong WC, West R. A Mobile App to Aid Smoking Cessation: Preliminary Evaluation of SmokeFree28. JMIR*.* 2015;17(1):e17.

7. van der Deen FS, Ikeda T, Cobiac L, Wilson N, Blakely T. Projecting future smoking prevalence to 2025 and beyond in New Zealand using smoking prevalence data from the 2013 Census. N Z Med J*.* 2014;127(1406):71-79.

8. Ministry of Health: Tobacco Use 2012/13: New Zealand Health Survey [Excel data files]. Wellington, New Zealand; 2014.

9. Stapleton J. Cigarette smoking prevalence, cessation and relapse. Stat Methods Med Res*.* 1998;7(2):187-203.

10. Etter JF, Stapleton JA. Nicotine replacement therapy for long-term smoking cessation: a meta-analysis. Tob Control*.* 2006;15(4):280-285.

11. McLeod M, Blakely T, Kvizhinadze G, Harris R. Why equal treatment is not always equitable: the impact of existing ethnic health inequalities in cost-effectiveness modeling. Popul Health Metr*.* 2014;12:15.
